# Allergy-unrelated eosinophil activation in the peripheral blood of children with neurodevelopmental disorders

**DOI:** 10.3389/fneur.2025.1680672

**Published:** 2025-11-19

**Authors:** Wenjing Ji, Muhan Li, Chenlu Yang, Yuanyuan Lu, Aimin Liang

**Affiliations:** Department of Children’s Health Care Center, Beijing Children’s Hospital, National Center for Children’s Health, Capital Medical University, Beijing, China

**Keywords:** neurodevelopmental disorders, eosinophil activation, allergic diseases, autism, spectral disorder, attention deficit and hyperactivity disorder

## Abstract

**Background:**

Neurodevelopmental disorders (NDDs) are increasingly associated with immune dysregulation, but eosinophil activation independent of allergic diseases remains unexplored in this population.

**Methods:**

Peripheral-blood eosinophil cationic protein (ECP) mRNA expression was quantified in 55 children with NDDs—including developmental delay (DD), autism spectrum disorder (ASD), attention deficit hyperactivity disorder (ADHD), communication disorder, and tic disorder—and 32 typically developing controls. Participants with allergies or recent infections were excluded.

**Results:**

The NDDs group exhibited significantly elevated ECP mRNA levels compared to controls, with median values of 165.87 copies/μL vs. 56.92 copies/μL (*p* < 0.001). Subgroup analyses confirmed increases in DD (*p* = 0.020), ASD (*p* = 0.002), and ADHD (*p* = 0.014), though no inter-subgroup differences were observed. Multivariate analysis identified NDDs as an independent predictor of ECP elevation (*p* = 0.048). Two high-ECP subjects harbored copy number variants affecting neuroimmune genes *ADA* and *LAT*. No correlations emerged between ECP levels and clinical behavioral scores.

**Conclusion:**

These findings establish a novel association between non-allergic eosinophil activation and NDDs, implicating neuroimmune crosstalk in disease pathogenesis and supporting ECP as a potential biomarker.

## Introduction

Neurodevelopmental disorders (NDDs) typically manifest in early childhood and are characterized by impairments in cognitive abilities, social interaction, communication, motor coordination and adaptive behaviors (1). Commonly, NDDs encompass a diverse array of conditions, including intellectual disability (ID), global developmental delay (GDD), communication disorders, autism spectrum disorder (ASD), attention deficit hyperactivity disorder (ADHD), tic disorder and specific learning disorders (2). When genetic or environmental factors disrupt the tightly coordinated events associated with important brain development processes, NDDs can occur (3). Recent research suggests that cellular immune dysregulation, shifted cytokine profiles, and immunogenetic variability are frequently observed in patients with NDDs and certain mouse models of NDDs (4, 5). Patients with NDDs also exhibit a higher incidence of immune related disorders, especially allergic diseases including food, skin (e.g., atopic dermatitis) and respiratory allergies (e.g., asthma, rhinitis), compared to children without these disorders (6–8).

There is a significant pathological and physiological convergence between allergies and NDDs. The activation, differentiation, mobility, and migration of eosinophils are intimately associated with the pathogenesis of allergic diseases (9, 10). Two recent large-scale epidemiological studies focusing on cohorts with eosinophilic esophagitis have reported a significant prevalence of NDDs at approximately 30% and ASD at around 21.59% (11, 12). This suggests that the dysregulation of eosinophil activity in neurodevelopment could be a potential pathogenic mechanism underlying NDDs. Despite a paucity of research into eosinophil mechanisms within the NDDs population, recent analyses of differentially expressed gene datasets derived from blood or serum samples of patients with schizophrenia (SCZ) have unveiled significant differences in gene expression profiles associated with eosinophils (13). These genetic variations may correlate with an increased risk of developing SCZ (14). Hence, the activation of eosinophils leads to the release of a multitude of highly cytotoxic granular proteins, cytokines, and chemokines, which can cross the blood–brain barrier (BBB) and alter neural functions, potentially playing a role in the development of NDDs (15, 16).

Human-specific eosinophil cationic protein (ECP), characterized by its potent extracellular cytotoxic functions, has been identified as a significant biomarker for eosinophil activation (17). The utility of ECP has been increasingly recognized, underscoring its essential role within the diagnostic, monitoring, and therapeutic frameworks for a diverse range of allergic conditions, with particular prominence in allergic asthma (18), allergic rhinitis (19), atopic dermatitis (20), and eosinophilic esophagitis (21).

In this study, we have conducted an examination of plasma ECP levels in children diagnosed with NDDs. To minimize the confounding effects of allergies, we have meticulously excluded children manifesting symptoms of respiratory, dermatological, and alimentary allergies. The study is designed to explore the potential link between eosinophils and NDDs, a critical endeavor for deepening our understanding of the underlying mechanisms of NDDs.

## Materials and methods

### Study participants and criteria

The study was conducted at the Children’s Health Care Center of Beijing Children’s Hospital, Capital Medical University, Beijing, China, from May 2022 to September 2023. Participants in the NDDs group were recruited from the clinic and either underwent a comprehensive diagnostic evaluation or received a re-evaluation at our center. The diagnosis was established by a multidisciplinary team that included at least two licensed child psychiatrists or developmental pediatricians with expertise in NDDs, in strict accordance with the Diagnostic and Statistical Manual of Mental Disorders, Fifth Edition (DSM-5) criteria. The DSM-5 requirement of clinically significant functional impairment across multiple settings (e.g., home and school) was applied to all diagnoses. To objectify the diagnostic process and quantify symptom severity, the following standardized assessments were utilized as clinically indicated: for children with DD, the Gesell Developmental Schedule (for children ≤6 years old) or the Wechsler Intelligence Scale (for children >6 years old) was used to obtain a comprehensive developmental quotient. In children with ASD, intellectual functioning was assessed using either the Gesell Developmental Schedule or the Wechsler Intelligence Scale, while core autistic behaviors were evaluated using the Autism Behavior Checklist (ABC) and the Clancy Autism Behavior Scale (CABS). For children with ADHD, the Swanson, Nolan, and Pelham Rating Scale-IV (SNAP-IV) was used to evaluate the severity of inattention and hyperactivity-impulsivity symptoms. This standardized diagnostic procedure was applied to all NDD conditions, including communication and tic disorders.

Children were excluded from both the NDDs and typically developing (TD) groups if there was evidence of allergic diseases. The exclusion was based on a two-tiered approach. First, the absence of clinically evident symptoms of allergic diseases, including respiratory allergies such as asthma and allergic rhinitis, skin allergies like atopic dermatitis, and food allergies, as confirmed by clinical interview and parental report. Second, objective laboratory testing for immediate allergic risk through serum food-specific IgE levels against common allergens like milk protein, egg white, wheat, soybeans, shrimp, and crab, with results detailed in Supplementary Table 1.

The TD group was composed of children recruited from local kindergartens and primary schools. To ensure the absence of neurodevelopmental or psychiatric concerns, all TD children were screened using the Chinese version of the Mini International Neuropsychiatric Interview for Children and Adolescents (MINI-KID), and no significant issues were identified. Furthermore, they were confirmed to have no neurological abnormalities, functional impairments, or histories of specialized clinical care for developmental or behavioral issues.

### Blood sample collection and processing

Blood samples were collected between 8:00 a.m. and 10:00 a.m., after a fasting period of at least 8 h but no more than 16 h, and with the child in a calm and resting state. For the purpose of this study, 2 mL of venous blood was collected from children in both the NDDs and TD groups using a serum separator tube. Serum was obtained by centrifuging blood at 3000 rpm for 10 min and subsequently used for the detection of food-specific IgE using the Food-specific IgE antibodies test kit (HOB, Jiangsu, China). Additionally, 1 mL of venous blood was collected in an EDTA anticoagulant tube and stored at −80 °C for the detection of ECP mRNA expression levels.

### ECP mRNA

Total RNA was extracted from whole blood samples using the Whole Blood Total RNA Kit (Simgen, Hangzhou, China) following the manufacturer’s guidelines. Reverse transcription and qPCR detection were performed in a single-step reaction using the ECP mRNA Detection Kit (fluorescence RT-PCR method) (Hangzhou Zheda Dixun Biological Gene Engineering, Hangzhou, China). Quantitative analysis of ECP mRNA levels was conducted on the SLAN®-The 96S fully automated medical PCR analysis system (Hongshi, Shanghai, China) using a TaqMan probe-based qPCR assay. The cycling parameters were as follows: an initial denaturation at 95 °C for 60 s, then 40 cycles of denaturation at 95 °C for 5 s, and annealing/extension at 60 °C for 30 s. The ECP gene-specific primers and TaqMan probe were: forward primer (5’-ACA GCT CAG AGA CTG GGA AAC-3′), reverse primer (5’-CCC ATA AGC CCC AAC AGA AG-3′), and probe (FAM)-TGG TTC CAA AAC TGT TCA CTT CCC-(BHQ1). A standard curve was generated using a template with a known ECP mRNA copy number for accurate quantification. The absolute copy number of ECP mRNA in the blood samples was determined by applying the test samples’ Ct values to the standard curve equation.

### Statistics

All statistical analyses were conducted using SPSS 23.0 software. Data that followed a normal distribution were examined with independent-samples t tests; data that departed from normality were examined with Mann–Whitney U tests and Spearman rank-order correlations. Linear regression models were used to examine the relationships among gender, age, NDDs status and ECP mRNA levels. All statistical tests were two-tailed with *p* < 0.05 considered significant.

## Results

### Demographic characteristics

Ultimately, children with allergic diseases were excluded from the study. The TD group comprised 32 children, with a gender ratio of 1:1 and an average age of 6.37 ± 2.49 years. The NDDs group included 55 children with the following diagnoses: 10 cases of DD, 21 cases of ASD, 21 cases of ADHD, 1 case of communication disorder, and 2 cases of tic disorder. The average age in the NDDs group was 5.71 ± 2.13 years, with a higher prevalence of boys over girls at a ratio of 1.9:1 (Table 1).

**Table 1:** Characterization of TD and NDDs groups (median [25th, 75th] or x ± SD).

Characteristic	TD group (*n* = 32)	NDDs group (*n* = 55)
Age (years)	6.37 ± 2.49	5.71 ± 2.13
Gender (*n*)		
Boy	16	23
Girl	16	12
DD *n* (%)	–	10 (18.2)
ASD *n* (%)	–	21 (38.2)
ADHD *n* (%)	–	21 (38.2)
CD *n* (%)	–	1 (1.8)
TID *n* (%)	–	2 (3.6)

NDDs: neurodevelopmental disorders; DD: developmental delay; ASD: autism spectrum disorder; ADHD: attention-deficit/hyperactivity disorder; CD: communication disorder; TID: tic disorder.

### Association between ECP mRNA expression and age and gender in TD children

**Figure 1:**
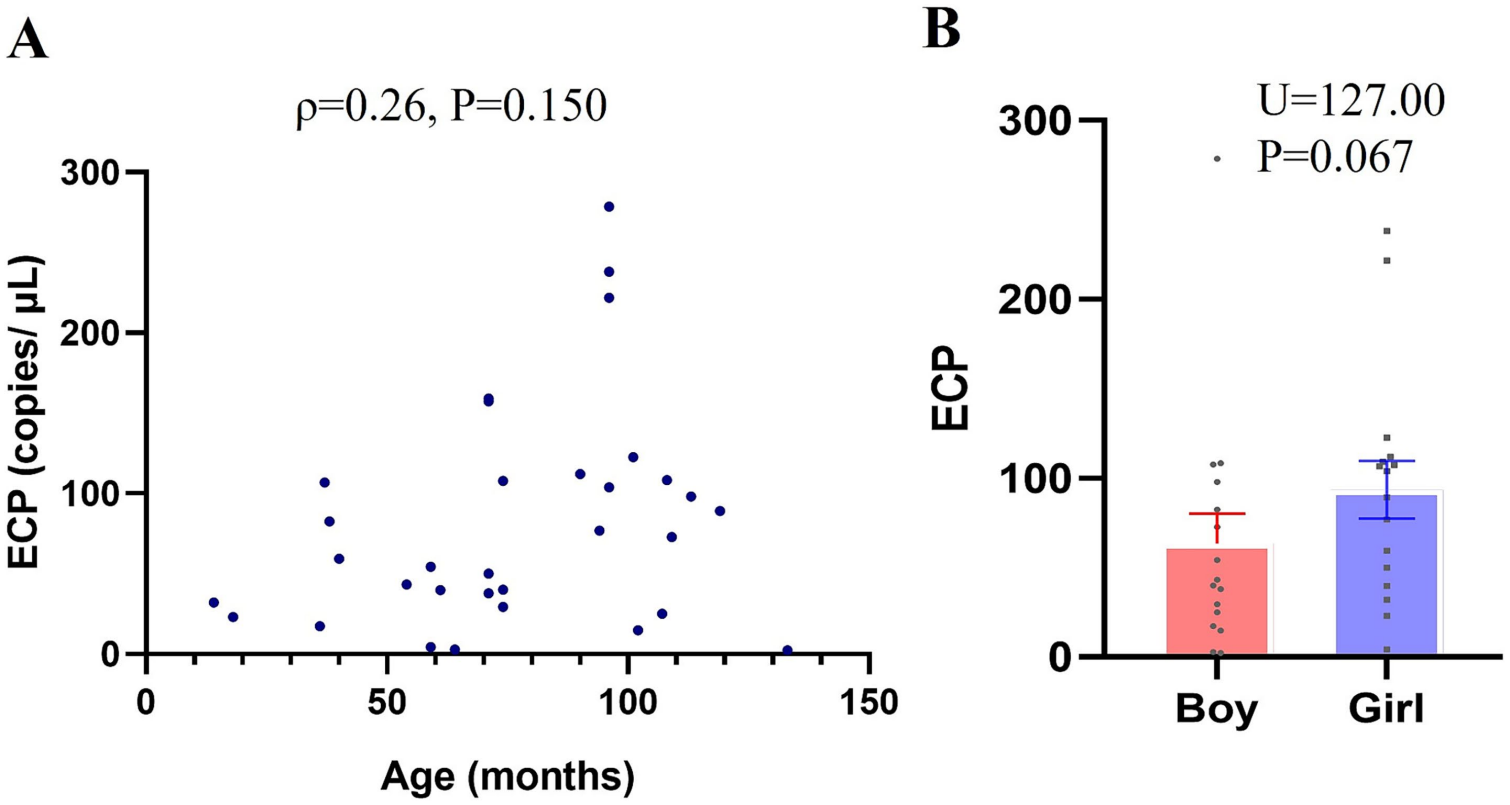
Association between ECP mRNA Expression and Age and Gender in Typically Developing (TD) Children. RT-PCR was utilized to measure ECP mRNA expression in peripheral-blood samples, with standard curve analysis determining copy numbers from Ct values. **(A)** Age-related differences in ECP mRNA expression within the TD group (scatter plot). **(B)** Gender-based comparison of ECP mRNA expression in the TD group. Data are presented as means ± SEM.

As illustrated in Figure 1, there was no significant correlation between ECP mRNA expression levels and age within the TD group (*ρ* = 0.26, *p* = 0.150). Additionally, no significant difference in ECP mRNA expression levels was observed between male and female children in the TD group (U = 177.00, *p* = 0.067).

### ECP mRNA expression level in NDDs and TD children

**Figure 2:**
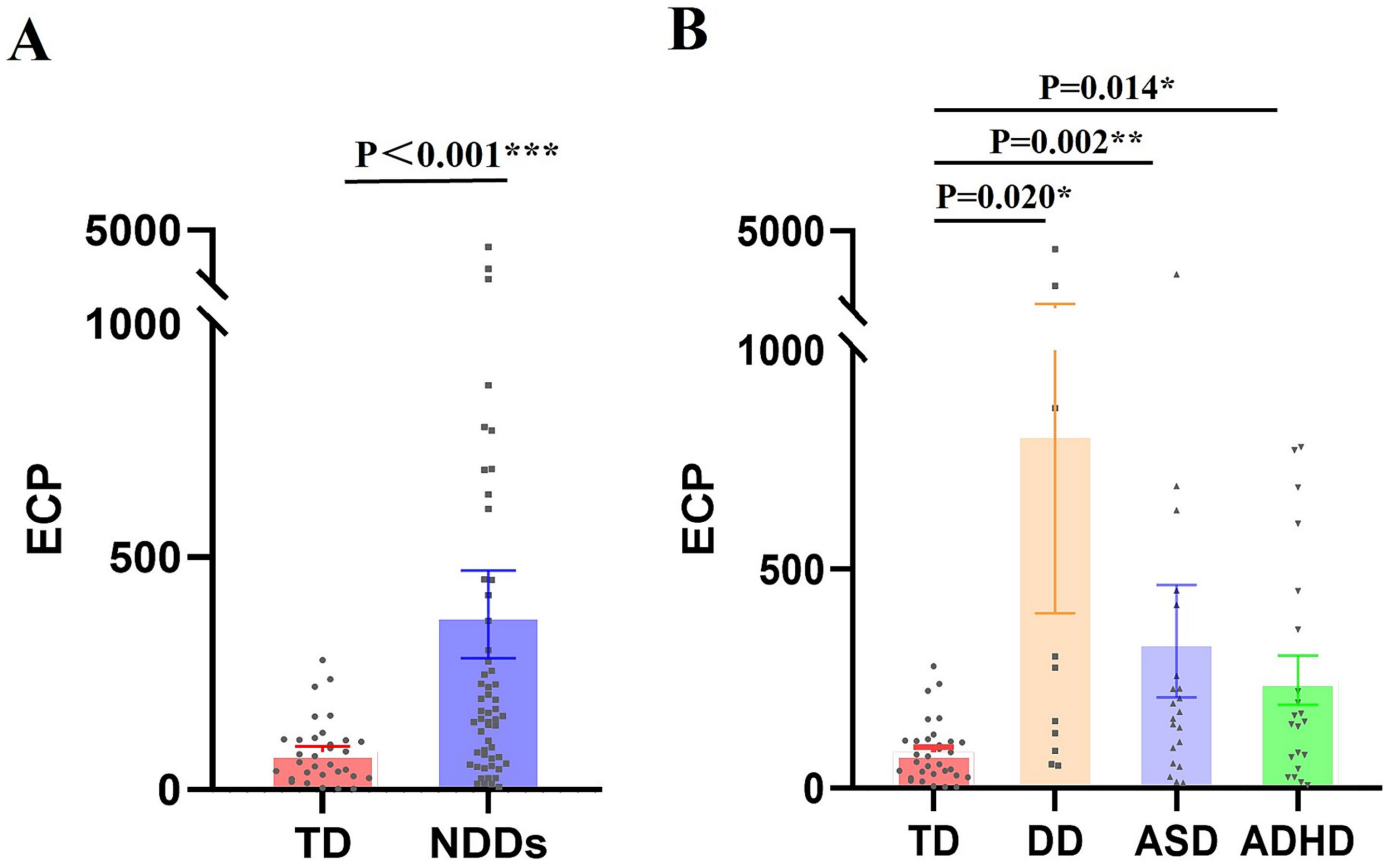
ECP mRNA Expression Levels in Children with NDDs compared to children in TD group. RT-PCR was utilized to measure ECP mRNA expression in peripheral-blood samples, with standard curve analysis determining copy numbers from Ct values. **(A)** ECP mRNA expression differences between the NDDs and TD groups. **(B)** Variability in ECP mRNA expression across different conditions within the NDDs group. Values are means ± SEM. *p < 0.05, **p < 0.01, ***p < 0.001.

As shown in Table 2, the peripheral-blood ECP mRNA expression level was significantly upregulated in children in the NDDs group compared with the TD group [median 165.87 vs. 56.92 copies/μL, *p* < 0.001]. Expression levels in children with DD, ASD, and ADHD were also significantly higher than those in the TD group: DD [median 214.16 copies/μL, *p* = 0.020], ASD [median 173.95 copies/μL, *p* = 0.002], and ADHD[median 165.87 copies/μL, *p* = 0.014]. However, there was no statistically significant difference in peripheral-blood ECP mRNA expression between the different disease groups within the NDDs (Figure 2).

**Table 2:** Comparison of serum ECP mRNA expression levels between NDDs subgroups and TD controls (median [25th, 75th]).

Group	*n*	ECP mRNA (copies/μL)	U	*p*
TD	32	56.92 (29.48, 106.79)	–	–
NDDs	55	165.87 (75.92, 450.54)	464.50	<0.001^**^
DD	10	214.16 (78.03, 1193.12)	59.00	0.002^**^
ASD	21	173.95 (74.25, 337.33)	169.00	0.002^**^
ADHD	21	165.87 (57.35, 527.02)	200.50	0.014^*^

ECP: eosinophil cationic protein; NDDs: neurodevelopmental disorders; TD: typically developing; DD: developmental delay; ASD: autism spectrum disorder; ADHD: attention-deficit/hyperactivity disorder.

*p < 0.05, **p < 0.01.

### Analysis of factors associated with ECP mRNA levels

The multiple linear regression model revealed a significant correlation between NDD status and ECP mRNA levels (*β* = −0.227, 95% CI: −533.020 to −1.860), while age (*β* = −0.033, 95% CI: −5.245 to 3.875) and gender (*β* = −0.049, 95% CI: −333.288 to 214.390) showed no significant correlation with ECP mRNA levels (Table 3).

**Table 3:** Correlational analysis of ECP mRNA levels.

Variable	β	95%CI	*p*
Gender	−0.049	(−333.288, 214.390)	0.667
Age	−0.033	(−5.245, 3.875)	0.766
NDDs status (Yes/No)	−0.227	(−533.020, −1.860)	0.048^*^

NDDs: neurodevelopmental disorders; CI: confidence interval.

^*^p < 0.05.

### ECP levels and clinical characteristics in NDDs group

Using 390.27 copies/μL (third quartile) as the threshold, we divided the NDDs group into High ECP (*n* = 13) and Non-high ECP (*n* = 42) groups. The High ECP group included 3 DD cases (23.1%), 5 ASD cases (38.5%), and 5 ADHD cases (38.5%), while the Non-high ECP group had 7 DD cases (16.7%), 16 ASD cases (38.1%), 16 ADHD cases (38.1%), 1 communication disorder case (2.4%), and 2 tic disorder cases (4.8%). There were no significant differences in disease composition between the groups (Table 4).

**Table 4:** Comparison of developmental and behavioral scale scores between high ECP group and non-high ECP group (median [25th, 75th] or x ± SD).

Scale	High ECP group (>390.27copies/μL)	Non-high ECP group (<390.27copies/μL)	t/Z	*p*
DD (*n* = 9)	*n* = 2	*n* = 7		
Gesell Adaptability Score	28, 76^a^	65 (62, 73)	N/A	N/A
ASD (*n* = 21)	*n* = 5	*n* = 16		
Gesell Adaptability Score	66.80 ± 16.23	59.00 ± 17.51	t = 0.870	0.397
ABC Score	53.80 ± 14.38	63.50 ± 15.10	t = −1.267	0.221
CABS Score	67 (52, 81.5)	59 (46, 69)	z = 57.500	0.142
ADHD (*n* = 21)	*n* = 5	*n* = 16		
Inattention	15.20 ± 2.95	14.25 ± 2.89	t = 0.639	0.530
Hyperactivity-Impulsivity	13.00 ± 4.42	12.63 ± 4.57	t = 0.161	0.874

DD: developmental delay; ASD: autism spectrum disorder; ADHD: attention-deficit/hyperactivity disorder; ABC: Autism Behavior Checklist; CABS: Clancy Autism Behavior Scale.

None of the comparisons reached statistical significance (*p* < 0.05).

^a^Data are presented as individual values due to the small sample size (*n* = 2).

No differences were observed in the Gesell adaptation score (66.80 ± 16.23 vs. 59.00 ± 17.51, *p* = 0.397), total CABS score (median 67 vs. 59, *p* = 0.142), and total ABC score (53.80 ± 14.38 vs. 63.50 ± 15.10, *p* = 0.221) among ASD children in the High and Non-high ECP groups. For ADHD children, no significant differences were noted in the total scores for attention deficit (15.20 ± 2.95 vs. 14.25 ± 2.89, *p* = 0.530) and hyperactivity (13.00 ± 4.42 vs. 12.63 ± 4.57, *p* = 0.874) on the SNAP-IV scale (Table 2).

Only two cases involving Trio-whole exome sequencing (Trio-WES) combined with copy number variant sequencing (CNV-seq) were tested, both from the High ECP group, and both yielded CNVs. One case (DD; ECP: 868.12 copies/μL) had a copy number deletion of approximately 3.78 Mb on the long arm of chromosome 20 at 20q13.12, described as seq [hg19] del (20) (q13.12) chr20: g.42601964-46386107del, involving genes such as *ADA* and *CD40*. The other case (DD; ECP: 778.99 copies/μL) had a duplication in the 16p11.2 region of chromosome 16, specifically seq [GRCh37/hg19] 16p11.2 (28803926-29,083,885) x3, involving genes such as *ATP2A1*, *CD19*, *TUFM*, and *LAT*.

## Discussion

We observed increased eosinophil activation in the peripheral-blood of children with NDDs, independent of allergic diseases. This association was consistent and significant across DD, ASD, and ADHD subgroups.

Our finding of elevated ECP in children with NDDs aligns with emerging evidence that eosinophils participate in brain function across the lifespan. Population-based studies now indicate that eosinophils and their cytotoxic products can influence cognitive and neurodevelopmental processes. For instance, in older adults without disability, lower eosinophil counts correlate with higher brain-derived neurotrophic factor (BDNF) levels and better performance in daily-living and concentration tasks (22). In Alzheimer’s disease, higher eosinophil counts and an elevated eosinophil-to-lymphocyte ratio are associated with early amyloid-*β* pathology (23). Longitudinal data from the UK Biobank further reveal that visceral adiposity contributes to declines in fluid intelligence in late-middle-aged women who have elevated eosinophil counts, whereas increased lean muscle mass appears to protect cognition by moderating eosinophil levels (24). Genetically, large-scale studies of schizophrenia have identified over 100 shared risk loci with concordant effect directions between eosinophil regulation and disease susceptibility (14). Together, these cross-disciplinary observations suggest that peripheral eosinophils not only reflect but may actively mediate brain health through lifestyle, pathological, and genetic pathways. Thus, our observation of non-allergic eosinophil activation in NDDs may represent a specific manifestation of a broader neuro-immune mechanism that influences neurodevelopment and cognitive function.

Although allergic diseases are more prevalent in ASD and ADHD (25, 26), the mechanisms linking them, potentially involving genetic and immune dysregulation (27, 28), remain unclear. While eosinophils are central to allergy pathogenesis (9, 10), their potential role as mediators between NDDs and broader immune dysregulation, including allergy, is understudied. Eosinophil-related pathways are also implicated in other psychiatric disorders such as schizophrenia (29) and depression (30, 31). Notably, CNV analysis in two NDD children revealed mutated regions encompassing the *ADA* gene, associated with both ASD and eosinophilia, and the *LAT* gene, crucial for brain development and amino acid transport regulation (32, 33). Significantly, the L-type amino acid transporter LAT1, influenced by *LAT*, is essential for activating Th2 cells that drive allergic eosinophilic inflammation (33). These genetic findings suggest potential drivers of eosinophil activation specific to NDDs.

This activation is regulated by key signaling pathways. Alterations in phosphatidylinositol 3-kinase-protein kinase B (PI3K-Akt) metabolism and its downstream effectors during early brain development may contribute to increased NDD prevalence (34). The mTOR pathway, pivotal for protein synthesis and brain homeostasis in autism, represents a potential link between immune disturbances and behavioral deficits (35). Dysregulation of Janus kinase/signal transducer and activator of transcription (JAK/STAT) signaling and cytokine profiles within the nervous system are further implicated in the pathogenesis of neurodegenerative diseases. (36). Histamine, proposed to explain allergy overrepresentation in ADHD (37), also directly stimulates eosinophil migration and mast cell recruitment (38).

Activated eosinophils exert cytotoxicity primarily through reactive oxygen species (ROS) release via membrane-bound NADPH oxidase 2 (NOX-2) and degranulation of cytotoxic proteins including major basic protein (MBP), ECP, and eosinophil-derived neurotoxin (EDN). They also produce cytokines such as IL-4 and IL-13, and lipid mediators (39, 40). Although the CNS is immunologically privileged by the BBB (41), this barrier can be compromised. The eosinophil chemotactic factor CCL11 can cross the intact BBB in mice, affecting neural circuits and behavior (42), and elevated levels correlate with reduced neurogenesis in mice and neurodegeneration in humans (15). The presence of eosinophils near neural cells in mice suggests involvement in gut-brain signaling (43). Critically, eosinophil-derived toxins, particularly MBP and EDN, cause tissue damage (16), and ECP demonstrates neurotoxic potential by inducing death in cerebellar granule neurons and astrocytes (44). This neurotoxicity disrupts synaptic integrity, induces oxidative stress, and promotes neuroinflammation with release of pro-inflammatory cytokines like IL-6 and TNF-*α* (45, 46), collectively impairing neurological function and disrupting CNS homeostasis in neuropsychiatric disorders.

Several limitations of this study warrant mention. First, the heterogeneity and uneven distribution of NDD subtypes in our cohort, which was predominantly composed of ASD and ADHD cases, may limit the generalizability of findings across all NDDs. Second, the subdivision of the NDDs group based on ECP levels yielded small and unbalanced subgroups, which likely reduced the statistical power for clinical correlation analyses and increased the risk of type II errors. Furthermore, the single-center design may affect the generalizability of our findings, and the observational nature of this study precludes causal inference. Future multi-center studies with larger, well-balanced samples and prospective or experimental designs are needed to validate these findings and elucidate the underlying mechanisms.

## Conclusion

There is a growing body of evidence suggesting that cells and molecules of the immune system play significant roles in neurodevelopment. Neuroimmune abnormalities are increasingly recognized as potential pathogenic mechanisms underlying NDDs. Collectively, our data suggest a link between NDDs and eosinophil activation, which warrants further investigation.

## Data Availability

The raw data supporting the conclusions of this article will be made available by the authors, without undue reservation.
