# Supplementary material for: Allergy-unrelated eosinophil activation in the peripheral blood of children with neurodevelopmental disorders
Source: Front Neurol. 2025 Nov 19;16:1680672. doi: 10.3389/fneur.2025.1680672 (PMC12672305; doi:10.3389/fneur.2025.1680672)
Supplement: Supplementary file 1 [file Table_1.DOCX]

**Supplementary Material**

| **Supplementary Table 1.** Comparison of the food specific IgE level between the TD and NDDs children | | | | |
| --- | --- | --- | --- | --- |
| Food | IgE | | | |
|  | TD group | NDDs group | *t* | *P* value |
| Milk | 0.20±0.31 | 0.32±0.39 | 1.449 | 0.151 |
| Egg | 0.327±0.62 | 0.36±0.83 | 0.527 | 0.597 |
| Wheat | 0.51±1.90 | 0.17±0.28 | 1.305 | 0.196 |
| Soybean | 0.14±0.61 | 0.23±1.35 | 0.370 | 0.712 |
| Shrimp | 0.03±0.05 | 0.04±0.09 | 0.776 | 0.440 |
| Crab | 0.01±0.04 | 0.02±0.07 | 0.631 | 0.530 |

Values are means ± SD.
